# Facing crises in the 21st century: microfluidics approaches for antibiotic discovery

**DOI:** 10.1111/1751-7915.13908

**Published:** 2021-08-01

**Authors:** Miguel A. Matilla

**Affiliations:** ^1^ Department of Biotechnology and Environmental Protection Estación Experimental del Zaidín Consejo Superior de Investigaciones Científicas Prof. Albareda 1 Granada 18008 Spain

## Abstract

We urgently need new antibiotics to counteract the rising in the emergence of multidrug‐resistant microorganisms. To improve the identification of antimicrobial compounds of microbial origin, numerous multidisciplinary approaches are being implemented. However, the development of innovative microbial cultivation strategies is necessary to exploit the full biosynthetic potential of non‐culturable microorganisms. Here, I highlight various articles that employ high‐throughput microfluidic‐based strategies to identify novel antimicrobial metabolites based on bacterial activities. The rapid development of this technology will likely advance the field of antibiotic discovery.

## Graphical abstract

New antibiotics are urgently needed. Here, I highlight various articles that employ high‐throughput microfluidic‐based strategies to identify novel antimicrobial metabolites based on bacterial activities.

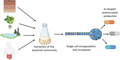

Currently, humanity is facing several major environmental and health problems. Amongst them are climate change, food shortage, soil health, pollution of marine and freshwaters, cancer and antibiotic resistance. In relation to the latter, two million people are annually infected with multidrug‐resistant microorganisms (MRMs) just in the United States (Dadgostar, 2019), and current estimations indicate that MRMs will cause 10 million deaths per year by 2050 if prompt actions are not undertaken (Trotter *et al*., 2019). This global health problem is mainly driven by the misuse of antibiotics, with an increased antibiotic consumption of 65% between 2000 and 2015 (Klein *et al*., 2018). It is even estimated that this figure will increase up to 200% in the 2015–2030 period if current consumption levels are maintained (Klein *et al*., 2018). This critical situation requires us not only to reduce the global antibiotic consumption but also to identify new classes of antibiotics with novel targets and mechanisms of action (Klein *et al*., 2018; Theuretzbacher *et al*., 2020; Udaondo and Matilla, 2020).

Secondary metabolites of microbial origin currently represent the main source of promising molecules with antimicrobial activities with agricultural and medical applicability (Newman and Cragg, 2016; Scherlach and Hertweck, 2021). However, the metabolic potential of most microorganisms remains largely unexplored (Medema *et al*., 2021). Notably, major difficulties to access the complete microbial biosynthetic potential are associated with the low culturability of environmental microbes under laboratory conditions, as well as the frequent crypticity, of gene clusters involved in the biosynthesis of secondary metabolites (Li *et al*., 2021; Medema *et al*., 2021; Scherlach and Hertweck, 2021). Hence, considerable progress is being made to develop and improve approaches based on microbial culturability, data mining, synthetic biology and analytical chemistry (Medema *et al*., 2021; Scherlach and Hertweck, 2021). For the former, one of these approaches includes the use of microfluidics‐based strategies.

Microfluidics strategies are becoming indispensable tools for microbiologists as they permit miniaturization, automation and integration of multi‐step experiments. Furthermore, they facilitate: (i) the cultivation of microorganisms under a great variety of experimental conditions; (ii) the analysis of the microbial biodiversity of clinical and environmental samples; (iii) the screening of combinations of drugs to analyse their effectiveness against MRMs; (iv) the analysis of interactions between biological molecules or (v) the identification of enzymes with activities of interest (Mair *et al*., 2017; Scheler *et al*., 2019; Pérez‐Rodríguez *et al*., 2021). A recent report in *Microbial Biotechnology* showed how high‐throughput microfluidics in combination with fluorescence‐activated cell sorting can overcome the current limitations of the viability of environmental microbes under laboratory conditions (Oberpaul *et al*., 2021). As such, this approach enabled complex microbial communities to be divided into single cells allowing individual strains to grow at their own rate without competing for space or nutrients with other microbes. Using this experimental strategy, the authors analysed the bacterial diversity of forest soil samples and examined the antimicrobial potential of 1071 microbial isolates from 57 different genera through their individual cultivation in agarose‐solidified microdroplets (~ 33 pL in volume, ~ 40 μm in size). To identify culture conditions that gained a broader microbial diversity, microorganisms were first encapsulated in different culture media and cultivation success (i.e. culturable microbial diversity in each growth condition tested relative to the original microbial diversity in the soil samples) was analysed by next‐generation sequencing (NGS). The total culturable microbial biodiversity was screened for bioactivity against relevant bacterial and fungal pathogens like *Mycobacterium tuberculosis* and *Zymoseptoria triciti*. Subsequently, bioactive organic extracts were analysed using the combination of ultra‐high‐performance liquid chromatography‐ultra‐high‐resolution mass spectrometry and molecular networking approaches. The most bioactive strains were found to belong to the *Pseudomonas*, *Erwinia* and *Streptomyces* genera, and their antimicrobial properties were associated with the biosynthesis of different non‐ribosomal peptides, polyketides and phospholipids, including massetolides, serratamolides, macrotetrolides and lysopalmitoyl‐phosphoethanolamine (Oberpaul *et al*., 2021).

In another recently published article in *eLife*, the authors aimed to analyse the antimicrobial activity of individual bacterial isolates from a complex soil microbial community. Using microfluidic droplets, they designed an integrated microbial cultivation and screening strategy (Mahler *et al*., 2021). To mimic environmental conditions from the isolation source, the medium for single‐cell microencapsulation (~ 200 pL in volume) and bacteria cultivation originated from the original soil and also contained plant‐derived metabolites and solid soil particles (< 40 μm in size). Droplets were surfactant‐stabilized in perfluorinated oil phase to reduce biological and chemical cross‐contamination between droplets or between droplets and the oil phase. Bacterial growth inside droplets was monitored using brightfield microscopy and the results revealed that in‐droplet cultivation enables a higher number of bacterial cells to replicate as compared to conventional agar plate cultivation. Additionally, based on NGS data analysis, a higher bacterial diversity was found in droplets in comparison with standard plate cultivation. Antibiotic production inside the droplets was screened by the injection of two fluorescent reporter strains, *Escherichia coli* and *Bacillus subtilis*, into these cultivation compartments. Previously to the injection of the reporter strains, the droplets were cultivated aerobically during an extensive period of time (approximately one month) to allow the bacterial colony to proliferate inside the droplet and to reach the metabolic state needed for antibiotic biosynthesis. Based on the fluorescence signal, those droplets containing isolates with high antibacterial activity (i.e. low fluorescence intensity) were selected *via* dielectrophoresis. The selected inhibiting bacterial isolates belonged to 13 different bacterial orders that included genera like *Bacillus, Microbacterium, Sphingomonas, Kocuria* and *Leifsonia*. To identify the antibiotic compounds produced by these strains, the authors selected one droplet isolate with strong antibacterial and antifungal properties against relevant human pathogens like *Pseudomonas aeruginosa*, *Mycobacterium vaccae* and *Candida albicans*. Using liquid chromatography coupled with high‐resolution mass spectrometry, they identified bacillaenes and surfactins as antimicrobial compounds (Mahler *et al*., 2021).

Despite the great efforts to mine microbial genomes to identify novel biosynthetic gene clusters and prioritize research aimed at novel antibiotic discovery (Medema *et al*., 2021; Scherlach and Hertweck, 2021), major difficulties remain to unlock the hidden biosynthetic potential of non‐culturable microbes. For instance, current estimates indicate that up to 99% of microorganisms cannot be cultured under laboratory conditions (Lok, 2015), which hinders the isolation of novel bioactive natural products. Therefore, new strategies are required to grow microbial isolates recalcitrant to cultivation. This highlight article aimed to emphasize the successful use of high‐throughput microfluidics approaches as a tool to discover novel compounds with antibiotic activities. Remarkably, given that secondary metabolites have been proposed to act as inter‐ and intrakingdom signalling molecules (van der Meij *et al*., 2017; Scherlach and Hertweck, 2020), microbial co‐cultivation methods are frequently used to activate the expression of silent bioactive metabolites (Herkersdorf *et al*., 2021; Scherlach and Hertweck, 2021). In this sense, microfluidics platforms are likely to facilitate the identification of novel bioactive molecules through the development of high‐throughput co‐culturing screenings.

## Conflict of interest

The author declares that there is no conflict of interest.
